# Parental bonding and personality characteristics of first episode intention to suicide or deliberate self-harm without a history of mental disorders

**DOI:** 10.1186/1471-2458-13-421

**Published:** 2013-05-01

**Authors:** Ya-Fen Hsu, Po-Fei Chen, For-Wey Lung

**Affiliations:** 1 Department of Psychiatry, Kaohsiung Armed Forces General Hospital, Kaohsiung, Taiwan; 2 Department of Internal Medicine, Kaohsiung Armed Forces General Hospital, Kaohsiung, Taiwan; 3 Calo Psychiatric Center, Pingtung County, Taiwan; 4 Taipei City Psychiatric Center, Taipei City Hospital, No.309, Songde Rd., Xinyi Dist., Taipei City 11080, Taiwan; 5 Department of Psychiatry, National Defense Medical Center, Taipei, Taiwan

**Keywords:** Intention to suicide, Deliberate self-harm, Community, Parental bonding, Alexithymic trait, Structural equation modeling

## Abstract

**Background:**

There is substantial overlap between deliberate self-harm (DSH) and intention to suicide (ITS), although the psychopathologies and motivations behind these behaviors are distinctly different. The purpose of this study was to investigate (i) the pathway relationship among parental bonding, personality characteristics, and alexithymic traits, and (ii) the association of these features with ITS and DSH using structural equation modeling to determine the risks and protective factors for these behaviors.

**Methods:**

Sixty-nine first-time DSH and 36 first-time ITS patients without medical or psychiatric illnesses, and 66 controls were recruited. The Parental Bonding Inventory (PBI), Eysenck Personality Questionnaire (EPQ), 20-item Toronto Alexithymia Scale (TAS-20), and the Chinese Health Questionnaire (CHQ) were filled out by the participants.

**Results:**

Our structural equation models showed that parental bonding had the greatest influence on the development of DSH behavior in patients. On the other hand, participants who were younger, less extraverted, with a greater extent of the alexithymic trait of difficulty identifying feeling (DIF), and a worse mental health condition, were more likely to develop ITS behavior. Males were more likely than females to develop the alexithymic trait of DIF.

**Conclusions:**

Although there are many covariates that affect both ITS and DSH behaviors, these covariates may have different functions in the development of these behaviors, thus revealing the psychopathological difference between DSH and ITS. Policymakers should consider these differences and build intervention and prevention programs for gender- and age-specific high-risk groups to target the differences, with a focus on family counseling to treat DSH and a focus on attempting to increase emotional awareness to treat ITS.

## Background

The World Health Organization (WHO) has reported that suicide is among the 20 leading causes of death, and that approximately one million people die annually by suicide worldwide [1]. Given that a previous suicidal attempt is a major risk factor for completed suicide [2], the number of individuals who attempt suicide is even greater than the number of lives lost to suicide. Once a person has engaged in self-harm, he or she is 50 to 100 times more likely to die by suicide [3,4], and self-harm is also one of the strongest predictors of completed suicide [4]. Although substantial overlap exists between suicidal and non-suicidal self-harm, the motivations behind deliberate self-harm (DSH) and intention to suicide (ITS) are distinctly different [5]. The interpersonal-psychological theory of suicidal behavior (IPTS) [5] posits that to enact a suicide attempt, the person must have the desire to die and the capability to engage in a lethal attempt. Those who engage in DSH have no desire to die but have the ability to engage in a lethal attempt; in contrast, those with ITS have both the desire to die and the capability to engage in a lethal attempt [5]. There may be others who have suicidal ideation but no encouragement to engage in a lethal attempt. Therefore understanding the differences in etiology and risk factors between ITS and DSH can help medical professionals and policymakers to develop prevention and intervention programs that target these behaviors.

Given that the risk of suicide increases when a first-degree relative has committed suicide [6], and that self-harming behaviors can also run in families [7], there is a familial influence on both ITS and DSH. Perceived anomalous parenting styles have been found to be associated with suicidal behavior [8,9]; more specifically, a low level of perceived parental care and a high level of parental control were associated with a repetition of suicidal behavior [10]. Along the same lines, a lower level of care and a higher level of maternal overprotectiveness were found in those adolescents with suicidal ideation and those who had made suicide attempts [11]. Therefore familial factors, specifically the style of parenting, play a role in increasing the risk of suicidal ideation and attempts.

Besides parental bonding, personality has also been found to be associated with suicidal ideation. Among patients with depression, those with a higher level of neuroticism and lower levels of extraversion, openness, and conscientiousness were more likely to attempt suicide [12]. A Ukrainian study also found that extraversion is the most discriminating factor among the “Big Five” traits that most commonly distinguish control subjects from those who made suicide attempts, regardless of gender [13]. People who have difficulty identifying feelings (DIF), differentiating feelings, verbalizing feelings, and communicating feelings are depicted as having alexithymia [14]. Alexithymia has been defined as a stable personality characteristic, with some factors that depend on the overall mental health of the subject [15]. Alexithymia is negatively correlated with extroversion [16], and positively correlated with neurotic personality characteristics [17,18]. There is a high prevalence of alexithymia in those with mental disorders, especially depressive disorders [19]. As a factor of alexithymia, DIF can help to identify those with a high risk of mental distress [20]. Even though substantial overlap exists between DSH and ITS. However, aside from the differences in motivation to die that lie behind these behaviors [5], those with a tendency toward major depressive disorder and/or post-traumatic disorder have a greater intention to die than those with DSH behaviors only [21]. In contrast, those with DSH behaviors have a greater tendency toward borderline personality disorder than those without DSH behaviors [21]. Furthermore, in women with borderline personality disorder, the method of DSH can distinguish DSH and ITS [22]. Therefore, the presence of a mental health condition and particular methods of DSH can also increase the risk of DSH and ITS. Given that parental bonding, personality characteristics, and alexithymic traits have been found to influence the development of psychiatric disorders [20], personality characteristics and alexithymic traits might be proximal risk factors for psychiatric disorders and possible distal factors for DSH and ITS.

The purpose of this study was to investigate the pathway relationship among parental bonding, personality characteristics, alexithymic traits and their association with ITS and DSH using structural equation modeling (SEM) to determine the risks and protective factors for these behaviors in first-time ITS and DSH participants.

## Methods

### Participants

Participants were recruited from the emergency room of a teaching hospital in southern Taiwan. All participants were first-time ITS or DSH patients without a medical or psychiatric diagnosis. Sixty-nine DSH patients and 36 ITS patients were recruited. Another 66 patients were recruited from the chronic pain outpatient department as controls; they were without history of ITS, DSH or psychiatric disorders. All participants were interviewed by psychiatrists using the Mini-International Neuropsychiatric Interview (MINI) [23], which follows both the DSM-IV [24] and International Classification of Diseases–10 (WHO-ICD-10)[25] criteria for the diagnosis of psychiatric disorders; those meeting the criteria of a psychiatric diagnosis were excluded. A total of 171 participants were recruited. The protocol of the study was approved by the institutional review board of the teaching hospital in Taiwan, and is in accordance with the Declaration of Helsinki. Informed consent was obtained from the participants after detailed explanation of the purpose of the study.

### Materials

Demographic information was collected, and four self-report questionnaires—the Parental Bonding Inventory (PBI), Eysenck Personality Questionnaire (EPQ), 20-item Toronto Alexithymia Scale (TAS-20), and Chinese Health Questionnaire (CHQ)—were filled out.

### Parental bonding instrument (PBI)

The PBI is a 25-item self-report questionnaire that measures individuals’ recalled parental bonding during the first 16 years of life in the two dimensions of care and protection. It was developed by Parker, Tupling, and Brown in 1979 [26], and was translated into Chinese by Shu and colleagues in 1999 [27]. The care dimension measures parental care and involvement, versus rejection; and the protection dimension measures parental control and overprotection, versus promotion of autonomy. There are 12 items that measure the dimension of care and 13 items that measure protection, on a four-point Likert scale of “very unlike”, “unlike”, “like”, and “very like”, with scoring from 0 to 3. Higher scores in the care and protection dimensions reveal that patients perceive their parents to be more caring and/or protective. The PBI has demonstrated internal consistency (Chronbach’s alpha = 0.65–0.73) and reliability (test–retest reliability = 0.66–0.88) [27]. Our study found a Chronbach’s alpha of 0.88 for paternal care, 0.84 for paternal protection, 0.85 for maternal care and 0.84 for maternal protection. In this study, the control minus the protection variable was used.

### Eysenck personality questionnaire (EPQ)

The EPQ is a 25-item self-report inventory measuring the personality characteristics of extraversion and neuroticism. The EPQ was developed by Eysenck and Eysenck in 1975 [28], and Lu [29] established the Chinese version. There are 14 neuroticism items that measure an individual’s emotional dysfunction, and 11 extraversion items measuring an individual’s sociability. The participants were asked to respond to the items using “yes” and “no”, scoring 0 and 1 respectively. Higher scores in the neuroticism or extraversion dimensions revealed that the patient was more neurotic or extraverted. The Chinese version demonstrated a high internal consistency of 0.90 [29], with an internal consistency of 0.83 for the extraversion dimension and 0.80 for the neuroticism dimension [30]. Our study also found a high internal consistency of Chronbach’s alpha of 0.87 for the extraversion dimension and 0.90 for the neuroticism dimension.

### Toronto Alexithymia Scale (TAS-20)

The TAS-20 is a 20-item self-report inventory measuring alexithymia as a three-dimensional construct of DIF (7 items), difficulty describing feelings (DDF; 5 items), and externally oriented thinking (EOT; 8 items) [31]. The participants were asked to respond to these items on a five-point Likert-scale of “greatly disagree”, “disagree”, “no comment”, “agree”, and “greatly agree”, scoring from 1 to 5. The TAS-20 was developed by Taylor and colleagues in 1990, and modified into the Taiwanese version by Lin and Chan in 2003 [32]. The Taiwanese version of the TAS-20 showed good construct and alpha coefficients of 0.82, 0.70, and 0.47 for the DIF, DDF, and EOT dimensions, and an alpha coefficient of 0.84 for the overall scale [32,33]. This study resulted in internal consistency of Chronbach’s alpha of 0.92 for the DIF dimension, 0.74 for the DDF dimension and 0.54 for the EOT dimension. The EOT dimension of the TAS-20 have a lower internal consistency in studies in Taiwan. This is consistent with our previous study [20], and we believe this is due to the Asian cultural context of Chinese speakers being more somatic-oriented than those in the Western cultures [34], and EOT is a preferred method of emotional expression which fit within the Eastern cultural values [35]. Chen et al. [20] found that the DIF of the TAS-20 is an effective screening index for those at high risk of mental health conditions. A Turkish study found that family over involvement in other members' concerns, which might cause a lack of privacy, was related to young adults' DIF [36]. This phenomenon of living with extended families is also common in Taiwan. Furthermore, a Japanese study also found that adolescents with psychosomatic and/or behavioral problems scored significantly higher than normal adolescents on the DIF index [37], and the DIF dimension of alexithymia have found in numerous studies to be related to somatization [38-40].

### Chinese Health Questionnaire (CHQ)

The CHQ is a self-administered questionnaire for the screening of minor psychiatric disorders in the community or in non-psychiatric departments. The CHQ was modified from the General Health Questionnaire developed by Goldberg [41], and was modified by Cheng and Williams [42] into the 12-item brief psychiatric screening test designed for the Chinese culture. The participants were asked to respond to the 12 items on a four-point Likert scale of “not at all”, “same as usual”, “more than usual”, “a lot more than usual”, with the respective scores 0, 0, 1, and 1. Cheng et al. [43] demonstrated internal consistency values of 0.84 and 0.83 for the CHQ. Our study also demonstrated high internal consistency of 0.85.

### Statistical analysis

The data were analyzed using SPSS 15.0 (Chicago, IL) and AMOS 7.0 (SPSS) for Windows software packages. Descriptive analysis was performed using SPSS 15.0, and SEM was performed using AMOS 7.0.

The SEM results are presented as chi-square, p value, comparative fit index (CFI), and adjusted goodness-of-fit indices (AGFI). A model with a p value greater than 0.05, CFI values greater than 0.95, root mean square error of approximation (RMSEA) less than 0.08, and an AGFI greater than 0.9 implies that the null model approximates the real structure. For the SEM results, only the parsimonious models are shown, meaning that only statistically significant variables are presented.

## Results

Comparison of the demographic distribution among the DHS and ITS patients and the controls showed there were 32 (48.5%), 30 (43.5%), and 21 (58.3%) males in the control, DSH, and ITS groups respectively. The ages of the participants ranged between 14 and 86 years old, with mean ages of 50.13 (SD = 18.54), 43.25 (SD = 19.98), and 29.28 (SD = 11.17) years in the control, DSH, and ITS groups, respectively. The results of the chi-square test showed that there was no statistically significant difference in the demographic distribution of the three groups. However, a significant age difference was found among the three groups (F = 15.81, p < 0.001). Accordingly, age was controlled in the SEM analysis.

The results of the PBI, EPQ, and DIF scores of the TAS-20, and the CHQ, were compared among the DHS and ITS patients and the controls using ANOVA, as shown in Table 1. The parental bonding characteristics were assessed as the control minus the protection variable. The results for paternal and maternal bonding on the PBI were statistically significantly different among the three groups (F = 101.05, p < 0.001; F = 51.56, p < 0.001). In the paternal bonding dimension, significant differences were found between the DSH and ITS groups (DSH < ITS, p < 0.001), and the control and DSH groups (Control > DSH, p < 0.001). Furthermore, significant differences were found between maternal bonding of the DSH and ITS (DSH < ITS, p < 0.001), and the control and DSH groups (Control > DSH, p < 0.001). The extraversion and neurotic personality characteristics of the EPQ were also statistically significantly different among the three groups (F = 5.19, p = 0.007; F = 14.34, p < 0.001). Differences were found between the control and ITS groups (Control > ITS, p = 0.001), and the control and DSH groups (Control > DSH, p = 0.001) in the extraversion dimension; and between the control and ITS groups (Control < ITS, p < 0.001) and the control and DSH groups (Control < DSH, p < 0.001) in the neuroticism dimension. The DIF dimension of the TAS-20 and the CHQ were both statistically significantly different among the three groups (F = 14.45, p < 0.001; F = 13.19, p < 0.001, respectively). For the DIF dimension, differences were found between the control and ITS groups (Control < ITS, p < 0.001), and the control and DSH groups (Control < DSH, p < 0.001). In the CHQ, differences were also found between the control and ITS groups (Control < ITS, p < 0.001), and the control and DSH groups (Control < DSH, p < 0.001). This showed that the DHS and ITS patients and the controls differed in the level of parental bonding, as well as personality characteristics, alexithymic traits, and mental health.

**Table 1:** Comparison of the Parental Bonding Inventory (PBI), Eysenck Personality Questionnaire (EPQ), 20-item Toronto Alexithymia Scale (TAS-20), and Chinese Health Questionnaire (CHQ) among the three groups: deliberate self-harm (DSH), intention to suicide (ITS), and control participants (n = 171)

	**Control**	**DSH**	**ITS**		
	**(n = 66)**	**(n = 69)**	**(n = 36)**		
	**mean (SD)**	**mean (SD)**	**mean (SD)**	**ANOVA**	**Tukey post hoc test**
PBI					
Paternal control and protection	8.86 (8.62)	−10.18 (5.95)	0.94 (9.27)	F = 101.05	Control > ITS MD = −1.66 p = 0.705
				p < 0.001	DSH < ITS MD = 9.48 p < 0.001
					Control > DSH MD = 11.15 p < 0.001
Maternal control and protection	10.47 (9.52)	−3.62 (5.64)	7.28 (10.16)	F = 51.56	Control > ITS MD = 1.22 p = 0.801
				p < 0.001	DSH < ITS MD = 10.17 p < 0.001
					Control > DSH MD = 8.96 p < 0.001
EPQ					
Extraversion	7.62 (3.76)	6.47 (4.24)	4.95 (4.11)	F = 5.19	Control > ITS MD = −2.82 p = 0.001
				p = 0.007	DSH > ITS MD = −0.47 p = 0.858
					Control > DSH MD = 2.82 p = 0.001
Neuroticism	3.44 (3.34)	4.91 (3.55)	7.37 (3.89)	F = 14.34	Control < ITS MD = 4.56 p < 0.001
				p < 0.001	DSH < ITS MD = 0.12 p = 0.984
					Control < DSH MD = −4.44 p < 0.001
TAS-20					
Difficulty identifying feelings	17.00 (6.35)	19.83 (6.75)	24.47 (7.26)	F = 14.45	Control < ITS MD = 8.33 p < 0.001
				p < 0.001	DSH < ITS MD = 0.93 p = 0.782
					Control < DSH MD = −7.40 p < 0.001
CHQ	2.66 (2.87)	3.99 (3.34)	6.13 (3.77)	F = 13.19	Control < ITS MD = 3.87 p < 0.001
				p < 0.001	DSH < ITS MD = 0.41 p = 0.818
					Control < DSH MD = −3.46 p < 0.001

**Figure 1:**
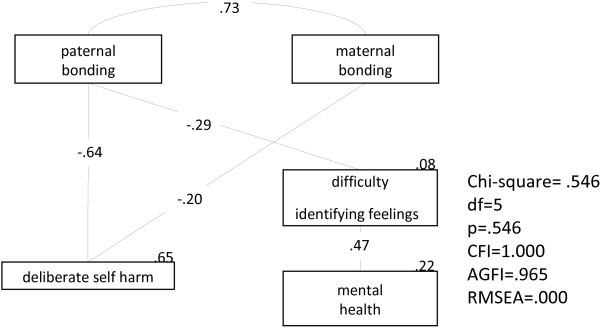
**Structural equation modeling of the pathway relationship among the Parental Bonding Inventory (PBI), Eysenck Personality Questionnaire (EPQ), 20-item Toronto Alexithymia Scale (TAS-20), and Chinese Health Questionnaire (CHQ) to compare the deliberate self-harm group and controls.** CFI: comparative fit index; AGFI: adjusted goodness-of-fit indices; RMSA: root mean square error of approximation.

In the SEM analysis, groups of participants were analyzed in pairs, the first of which comprised DHS patients and controls, and the second of which comprised ITS patients and controls. This was done to understand better how parental bonding, personality characteristics, alexithymic traits, and mental health contributed to the development of the behaviors of subjects with DHS or ITS when compared with the controls. The first SEM analysis, which examined the pathway relationship among parental bonding, personality characteristics, alexithymic traits, and mental health between the DSH group and controls, is shown in Figure 1. The model resulted in a p value of 0.546 (greater than 0.05), a CFI value of 1.000 (greater than 0.95), RMSEA of 0.000 (less than 0.08) and AGFI of 0.965 (greater than 0.9), thus showing that the null model approximates the real structure. The model showed that the factors directly associated with DSH were paternal bonding and maternal bonding (β = −0.64, p < 0.001; β = −0.20, p = 0.007). In this model, the DIF trait, mental health condition, and DSF accounted for 8%, 22%, and 65% of the variance, respectively.

**Figure 2:**
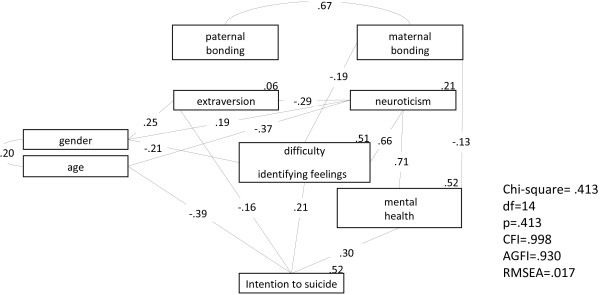
**The structural equation modeling of the pathway relationship among the Parental Bonding Inventory (PBI), Eysenck Personality Questionnaire (EPQ), 20-item Toronto Alexithymia Scale (TAS-20), and Chinese Health Questionnaire (CHQ) to compare the intention to suicide group and controls.** CFI: comparative fit index; AGFI: adjusted goodness-of-fit indices; RMSA: root mean square error of approximation.

The model used to compare the ITS group and the controls with respect to parental bonding, personality characteristics, alexithymic traits, and mental health resulted in a p value of 0.413, CFI value of 0.998, RMSEA of 0.017, and AGFI of 0.930, thus showing a good fit (as shown in Figure 2). The model showed that the factors directly associated with ITS behavior included age, extraverted personality characteristics, alexithymic DIF traits, and mental health (β = −0.39, p < 0.001; β = −0.16, p = 0.014; β = 0.21, p = 0.004; β = 0.30, p < 0.001) Younger participants, those who were less extraverted, and those with higher DIF alexithymic traits and worse mental health are most likely to develop ITS behavior. In this model, extraversion, neuroticism, DIF traits, mental health, and ITS accounted for 6%, 21%, 51%, 52%, and 52% of the variance, respectively.

## Discussion

Our study compared ITS and DSH participants with controls to investigate the differences between ITS and DSH. Our SEMs showed that parental bonding had the greatest influence on the development of DSH behavior, with paternal bonding having a greater influence than maternal bonding; neither DIF nor mental health had a direct influence on DSH. On the other hand, parental bonding did not have a direct influence on ITS; instead, an indirect effect of maternal bonding on ITS was found through the mediating factor of the alexithymic trait of DIF. Participants who were younger, less extraverted, and with a higher DIF alexithymic trait and worse mental health were more likely to develop ITS behavior. In these models, given that DSH accounted for 65% of the variance and ITS accounted for 52% of the variance, it can be concluded that DSH and ITS differ psychopathologically.

No demographic differences were found between the DSH and the control group; on the other hand, two demographic factors, age and gender, were directly or indirectly associated with ITS. Our results showed that age was directly associated with ITS; those who were older were less likely to show ITS behavior. In Taiwan, suicide has been reported to be the second most common cause of death among adolescents (15–24 year olds) [44]. This shows that adolescents have the highest risk of ITS in Taiwan, although they are younger than subjects included in our sample. In addition to age, gender had an indirect association with ITS through the mediating factor of the DIF factor of alexithymia. Males were more likely to be alexithymic (as shown in Figure 2). This is consistent with a previous study, which found males had higher scores for alexithymic traits than females [45]. Therefore, those who are male and have difficulty identifying their feelings, and those who are younger, are at higher risk of developing ITS behaviors than other members of the population.

Beside demographics, parental bonding was found to be associated with both ITS and DSH. The bonding to both parents was directly associated with DSH, and maternal bonding was indirectly associated with ITS through the mediating factor of the alexithymic trait of DIF. An influence of parental bonding on suicidal behavior was also found in previous studies, which showed that parental bonding was associated with the repetition of suicidal behavior [10], and with suicidal ideation and attempts in adolescents [11]. Alexithymics have recalled a parenting style of either overprotection or low care, which showed an association between parental bonding and alexithymic traits [46,47]. A similar pathway, with personality characteristics being the mediating factor between parental bonding and alexithymic traits, has also been found [20]. Furthermore, our study revealed, further, that paternal bonding had a greater association with DSH than maternal bonding. However, only maternal bonding had an indirect association with ITS; paternal bonding was not associated with ITS. Therefore, although parental bonding influences the development of both ITS and DSH behaviors, the role of paternal bonding is of primary importance in the development of DSH behavior, whereas the role of maternal bonding is of primary importance in the development of ITS behavior.

In addition to the level of DIF, ITS patients also had worse mental health. However, mental health did not influence the development of DSH behaviors. Depression has been associated with suicidal ideation [48]; feeling hopeless was determined to be a predictor of both suicidal ideation and depression [24]. In adolescents, a high association between depression and anxiety was associated with self-harming behavior in a follow-up cohort study [49]. However, this association was not found in our study in participants aged between 29 and 50 years old. Furthermore, those with intention to commit suicide were not excluded from the study of Moran and colleagues [49]. Given that ITS patients have statistically significantly worse mental health conditions than controls, public and medical health professionals should pay careful attention to patients who have attempted suicide, and refer them for psychiatric help when needed.

The first limitation of this study is related to the fact that a previous study showed that the vast majority of adolescents with DSH behavior do not seek medical help [50]. Given that the participants in this study were recruited from the emergency room of a hospital, our results can only be generalized to those who have sought help at a hospital. Second, the participants in this study were recruited from the emergency room of a general hospital in southern Taiwan. The rejection rate for recruitment was high when there were no medical rapport. In addition, given that we were unable to follow up those who rejected participation, the demographics of those who agreed to participate could not be compared with the demographics of those who declined to participate. Therefore, the external validity of the results needs further investigation. Lastly, Chen et al. [51] showed that the media tends to underreport mental illness as a reason for suicide in men in Taiwan. In India, the National Crime Records Bureau underestimates suicide in men by at least 25%, and in women by at least 35% [52]. This shows that some gender and cultural factors are associated with the tendency to commit suicide. Accordingly, semi-structured interviews regarding the motivation behind their behavior were conducted with all the patients who agreed to participate to determine whether they had intended suicide or self harm. However, we cannot eliminate the possibility that they underreported their intention to commit suicide. The atmosphere of the emergency room department is generally noisy and chaotic in Taiwan. We suggest in future studies a private room maybe used for these interviews to elevate medical rapport and diminish the affect from the environment. Notwithstanding those limitations, the strength of this study is that it included only first-time DSH and ITS participants without any psychiatric disorder or general medical disorders. Psychiatric inpatients, general medical patients, and community residents who attempt suicide have different psychological risk factors [53]. Therefore, this study excluded psychiatric disorders and other general medical disorders, and focused only on those in the community.

## Conclusions

Our study found that, although many covariates affect both ITS and DSH behaviors, these covariates may have different functions in the development of these behaviors, thus showing that DSH and ITS differ psychopathologically. Therefore, policymakers should take note of these differences and build intervention and prevention programs to target them, with prevention of DSH focused on family counseling, and prevention of ITS focused on increasing emotional awareness and targeting gender- and age-specific high-risk groups. Furthermore, given that those who show ITS behaviors have statistically significantly worse mental health condition than controls, public health and medical health professionals should screen for those who need further psychiatric attention and refer them for psychiatric help if needed.

## Competing interests

All authors have no conflict of interest to declare.

## Authors’ contributions

YFH and FWL contributed to the conception and design of the study. YFH oversaw the data collection process. FWL and PFC analyzed and interpreted the results. PFC wrote the draft of the manuscript. All authors modified and approved the final manuscript.

## Pre-publication history

The pre-publication history for this paper can be accessed here:

http://www.biomedcentral.com/1471-2458/13/421/prepub
